# Modelling the Genetic Risk in Age-Related Macular Degeneration

**DOI:** 10.1371/journal.pone.0037979

**Published:** 2012-05-30

**Authors:** Felix Grassmann, Lars G. Fritsche, Claudia N. Keilhauer, Iris M. Heid, Bernhard H. F. Weber

**Affiliations:** 1 Institute of Human Genetics, University of Regensburg, Regensburg, Germany; 2 University Eye Hospital Würzburg, Würzburg, Germany; 3 Institute of Epidemiology and Preventive Medicine, University Hospital Regensburg, Regensburg, Germany; 4 Institute of Genetic Epidemiology, Helmholtz Zentrum München, German Research Center for Environmental Health, Neuherberg, Germany; Innsbruck Medical University, Austria

## Abstract

Late-stage age-related macular degeneration (AMD) is a common sight-threatening disease of the central retina affecting approximately 1 in 30 Caucasians. Besides age and smoking, genetic variants from several gene loci have reproducibly been associated with this condition and likely explain a large proportion of disease. Here, we developed a genetic risk score (GRS) for AMD based on 13 risk variants from eight gene loci. The model exhibited good discriminative accuracy, area-under-curve (AUC) of the receiver-operating characteristic of 0.820, which was confirmed in a cross-validation approach. Noteworthy, younger AMD patients aged below 75 had a significantly higher mean GRS (1.87, 95% CI: 1.69–2.05) than patients aged 75 and above (1.45, 95% CI: 1.36–1.54). Based on five equally sized GRS intervals, we present a risk classification with a relative AMD risk of 64.0 (95% CI: 14.11–1131.96) for individuals in the highest category (GRS 3.44–5.18, 0.5% of the general population) compared to subjects with the most common genetic background (GRS −0.05–1.70, 40.2% of general population). The highest GRS category identifies AMD patients with a sensitivity of 7.9% and a specificity of 99.9% when compared to the four lower categories. Modeling a general population around 85 years of age, 87.4% of individuals in the highest GRS category would be expected to develop AMD by that age. In contrast, only 2.2% of individuals in the two lowest GRS categories which represent almost 50% of the general population are expected to manifest AMD. Our findings underscore the large proportion of AMD cases explained by genetics particularly for younger AMD patients. The five-category risk classification could be useful for therapeutic stratification or for diagnostic testing purposes once preventive treatment is available.

## Introduction

Age-related macular degeneration (AMD) is a common degenerative disease of the central retina and a leading cause of severe vision impairment in Western societies [1]. Advanced forms of AMD (late-stage AMD) are known as geographic atrophy (GA) of the retinal pigment epithelium (RPE) or neovascular (NV) complications with RPE detachment, scar formation, and subretinal hemorrhage [2], [3]. To date, effective therapeutic intervention is available for active NV, while GA still remains untreatable [4], [5].

AMD is a complex disease influenced by genetic and environmental factors with estimates of heritability varying from 45% to 71% [6]. So far, several AMD susceptibility loci have been identified. Two loci are accounting for an estimated 50% of AMD cases: complement factor H (*CFH*) on 1q32 and age-related maculopathy susceptibility 2 (*ARMS2*)/HtrA serine peptidase 1 (*HTRA1*) on 10q26 [7], [8]. Fine-mapping studies and functional analyses at the *CFH* locus indicate at least three independent risk variants [8]–[13]. At the *ARMS2/HTRA1* region, a single risk haplotype was found to fully explain the observed association [14].

A crucial role of the complement system in AMD pathogenesis was further supported by subsequent candidate gene studies. These studies identified risk-associated variants in or near three additional complement genes including the complement component 2 (*C2*)/complement factor B (*CFB*) [15], complement component 3 (*C3*) [16], [17] and complement factor I (*CFI*) [18]. In addition, variants in genes involved in the cholesterol and lipid metabolism were also implicated in AMD susceptibility [19], [20]. Strongest signals peaked near the hepatic lipase gene (*LIPC*) on chromosome 15q22 [19], [20], the cholesterylester transfer protein (*CETP*) and the lipoprotein lipase precursor (*LPL*) genes [19]. Also, among the most replicated AMD risk variants are two coding SNPs in the apolipoprotein E (*APOE*) gene [21], [22]. A recent genome wide association study established a significant association of AMD with rs9621532, a variant intronic to synapsin III (*SYN3*) and approximately 100 kb upstream of the tissue inhibitor of metalloproteinases-3 gene (*TIMP3*) [19]. Finally, common variations near VEGFA and FRK/COL10A1 were associated with AMD, further implicating angiogenesis as well as extracellular matrix metabolism in AMD pathogenesis [23].

To predict the genetic risk in complex diseases, testing of single susceptibility variants is generally of limited value [24]. In contrast, genotyping and evaluating a series of independent disease associated variants, a process also known as genetic profiling, may be more appropriate [24]. This can be facilitated by a genetic risk score (GRS) which could simply represent the sum of risk associated variants found in each individual. However, such an approach may not be particularly effective in the presence of greatly differing effect sizes of the respective variants [25]. Therefore, an extension to this model weighs each additional risk allele by its effect size. For example, Seddon et al. (2009) calculated a risk score for AMD based on 6 known genetic risk variants and additional environmental factors. Their model revealed good discriminatory power with a reported area-under-curve (AUC) of the receiver-operating characteristic of 0.82 [26]. Other studies reporting a GRS [19], [20], [23] primarily aimed at identifying novel variants without using independent data or a cross-validation approach and are thus likely biased to overestimate the effect of these variants. The quantification of the genetic risk based on frequently replicated AMD loci in a single study which is independent from locus identification is still lacking.

Here, we present a genetic risk model for AMD, specifically the late-stage forms of AMD, based on a large and well characterized AMD case-control study group including 986 cases and 796 controls. We selected 13 genetic variants from eight gene loci that have repeatedly been shown to be associated with AMD and computed a genetic risk score. This was used to establish a classification system that allows for discriminating subjects at high and low genetic risk. Environmental variables such as smoking or diet were not included in the model building.

## Results

### SNP selection based on published data and linkage disequilibrium structure

Eight loci (CFH, ARMS2/HTRA1, CFI, CFB, C3, APOE, LIPC and TIMP3) with 13 SNPs and established association with AMD were included into our genetic risk score modeling (**Table S1**). There were three further SNPs with reportedly established association, which we did not select for the model: (i) at the CFH locus, an association of four variants with AMD is known (rs1410996, rs800292, rs1061170, rs6677604); however, rs1410996 is present on two distinct haplotypes, each of which is tagged by rs800292 (correlation r^2^ = 0.473 to rs1410996 [27]) or rs6677604 (r^2^ = 0.283 to rs1410996 [27]), respectively [13], while rs800292 and rs667604 are uncorrelated (r^2^ = 0.008 [27]), (ii) among the three highly correlated ARMS/HTRA variants (rs10490924, rs11200638, and c.del443ins54; pairwise r^2^ = 1), rs10490924 was reported to fully capture the disease risk at this locus [28]. We therefore selected rs1061170, rs800292 and rs667604 at CFH and rs10490924 at the ARMS2/HTRA1 locus yielding the 13 SNPs for model building.

### Genotyping of SNPs in the Lower Frankonian AMD case-control study

We genotyped the selected 13 SNPs as well as the three highly correlated SNPs (to validate the correlations) in 986 cases and 796 controls from the Lower Frankonian AMD case-control study (**Table 1**). All variants showed high genotyping quality with an average call rate >99.5%. With the exception of rs1061170 at CFH, all genotypes were in Hardy-Weinberg equilibrium in controls (HWE, p>0.04). The variant rs1061170 was genotyped twice with two independent assays yielding identical genotypes and therefore persistent HWE violation in controls (p = 0.002) [29]. There were no missing genotypes at the 13 variants for any individual in the study.

**Table 1:** Summary characteristics of the case-control study.

	Cases	Controls	Total
Subjects	986	796	1782
GA^1^	229	-	
NV^2^	581	-	
Mixed GA+NV^3^	176	-	
Mean Age (S.D.) [in years]	78.7 (6.5)	78.3 (5.1)	78.5 (5.9)
Men [%]	34.1	39.3	36.4
Fraction smoker [%]^4^^,^ 5	15.9	14.3	

1 Geographic atrophy.

2 Neovascular AMD.

3 Mixed GA+NV: GA and NV in the same eye or GA in one and NV in the second eye.

4 Smoking was defined as ever smoked more than 20 pack years.

5 This variable was surveyed incompletely in cases and controls and thus was not further considered in the analysis.

### Association of the selected 13 SNPs with AMD

For each SNP, association with AMD was computed using a logistic regression model, unadjusted for age or gender (**Table 2**). Sensitivity analysis additionally adjusting for age and gender yielded similar results. Odds ratio (OR) estimates per AMD risk increasing variant ranged from 1.14 [95% CI: 1.00–1.30] for rs2285714 to 3.13 [95% CI: 2.68–3.68] for rs10490924 and were significantly different from unity for all 13 variants demonstrating sufficient statistical power in our study (**Table 2**). In a subgroup analysis, AMD cases with GA (n = 229) or NV (n = 581) or mixed GA+NV in one or both eyes (n = 176) were compared to controls using logistic regression for each variant separately (**Figure S1)**.

**Table 2:** Association results for the 13 known AMD associated variants in the lower Frankonian case-control study (986 cases, 796 controls) using single logistic regression.

									Frequency of risk allele in			
Nearby gene(s)	Marker	ID	Impact/effect of variant	Odds ratio	95% CI^1^	P-value^2^	Non risk allele	Risk allele^3^	Controls (N = 796)	Cases (N = 986)	AUC^4^ of variant	correlation^5^
*CFH*	rs1061170	1	p.Y402H	2.74	2.36–3.18	1.66E−45	T	C	0.365	0.600	0.676	
	rs800292	2	p.I62V	2.43	2.02–2.92	6.95E−23	A	G	0.761	0.888	0.606	0.150
	rs6677604	3	proxy for ΔCFHR3/CFHR1	2.19	1.82–2.64	1.42E−17	A	G	0.777	0.884	0.590	0.203
*ARMS2*	rs10490924	4	p.A69S	3.13	2.68–3.68	7.97E−54	G	T	0.189	0.441	0.684	
*CFB*	rs4151667	5	p.L9H	2.82	1.90–4.28	1.41E−07	A	T	0.951	0.982	0.530	
	rs438999	6	proxy for rs641153 (p.R32Q)	2.31	1.73–3.11	5.75E−09	C	T	0.915	0.962	0.542	0.01
*C3*	rs2230199	7	p.R102G	1.52	1.29–1.80	4.71E−07	G	C	0.175	0.245	0.556	
*APOE*	rs7412	8	p.R158C	1.41	1.12–1.80	0.003613	C	T	0.079	0.107	0.526	
	rs429358	9	p.C112R	1.35	1.09–1.69	0.006812	C	T	0.881	0.908	0.528	0.783
*PLA2G12A*	rs2285714	10	synonymous exonic, unknown	1.14	1.00–1.30	0.04839	C	T	0.409	0.443	0.523	
*LIPC*	rs493258	11	intergenic (36 kb upstream)	1.18	1.04–1.35	0.01277	T	C	0.538	0.580	0.531	
	rs10468017	12	intergenic (46 kb upstream)	1.26	1.08–1.46	0.002992	T	C	0.707	0.751	0.536	0.367
*SYN3/TIMP3*	rs9621532	13	intronic, unknown	1.58	1.09–2.30	0.01246	C	A	0.96	0.974	0.512	

1 CI = confidence interval.

2 P-values were derived from a logistic regression model with one SNP as covariate.

3 Risk allele is the allele that is associated with increased risk of AMD.

4 AUC = area-under-curve of the receiver-operating characteristic.

5 r^2^ values representing the correlation with the first SNP in each gene/locus based on 1000 genomes data (build 1) or HapMap release 22 [27].

### Computing the genetic risk score

**Figure 1:**
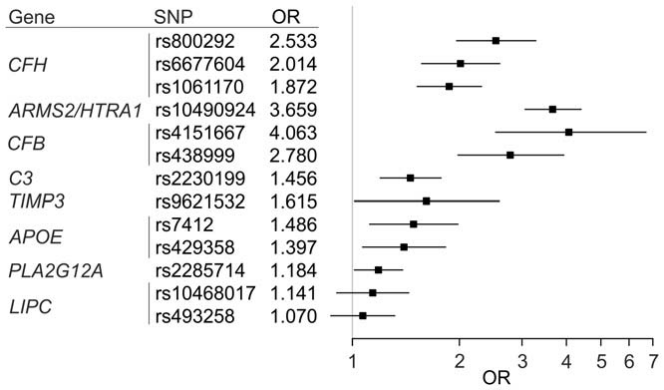
Risk estimates for each of thirteen AMD risk variants from eight gene loci. Odds ratios (OR) per risk allele were derived from multiple logistic regression models. Horizontal lines indicate 95% confidence intervals.

Based on the data from the 13 SNPs, we fit a multiple logistic regression model (**Figure 1**). The odds ratios in this model ranged from 1.070 to 4.063. This is, to our knowledge, the first study to report these 13 variants together in one multiple logistic regression accounting for other AMD risk variants. We computed a GRS for each individual as the sum of AMD risk increasing alleles weighted by the relative effect size of each SNP from the logistic model. We added the alpha estimate of −10.13 to center the GRS on zero for our study (see Methods). Cases had a significantly higher mean GRS (1.61, 95% CI: 1.53–1.69) compared to controls (−0.03, 95% CI: −0.12–0.06, p<0.01). The relative risk of AMD per GRS unit approximated by the OR was 2.72 (95% CI: 2.46–3.01). The mean GRS of our controls was slightly lower than the one for the HapMap data representing a general population (0.00, 95% CI: −0.14–0.14), which is in-line with our controls being selected for having no AMD.

### Good discriminative ability of the GRS

**Figure 2:**
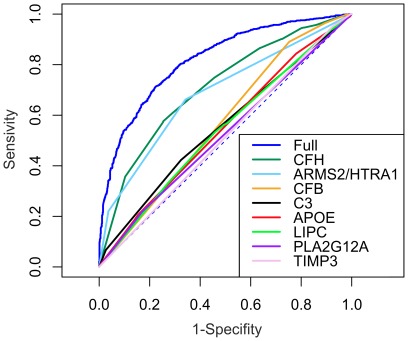
Area-under-the-curve of the receiver operating characteristic for the 13-SNP genetic risk score and by gene locus. Observed AUC was 0.820 and the locus-specific AUCs were 0.513, 0.524, 0.536, 0.547, 0.555, 0.571, 0.686 and 0.710 from bottom to top.

Computing the area-under-the-curve (AUC) of the receiver-operating characteristic for the 13-SNP GRS, we observed good ability to correctly classify those with and without the disease (AUC = 0.820, **Figure 2**). We also computed the AUC per locus demonstrating that the impact by gene varied substantially, as expected. The three SNPs at the CFH locus alone (rs800292, rs1061170, rs6677604) showed the highest classification efficiency (AUC = 0.710), followed by rs10490924 at ARMS2/HTRA1 (AUC = 0.684), and the remaining variants (AUC from 0.512 to 0.571) (**Figure 2**).

Although we specifically avoided selecting the SNPs based on association in our own data set but rather from the literature, there could be a potential overestimation of the AUC: We estimated the effect sizes per variant from our data and used these as weights for the GRS. To evaluate this potential over-estimation, we performed a sensitivity analysis via a cross-validation approach by repeated (i = 2000) random sub-sampling with 2/3rd of the data for model building and 1/3rd for testing. The cross validated AUC of 0.813 (95% CI: 0.813–0.814) is close to the one described in our initial study (AUC = 0.820).

### Developing a parsimonious genetic risk score model

We evaluated whether a parsimonious model based on our data could be developed. We thus explored several models by subsequently excluding the loci with the weakest AUC and found a model restricted to 10 variants with equally discriminatory ability (AUC 0.820) and equal model fit (R^2^ = 0.247) (**Table 3**). This model could be of value for translational studies minimizing the genotyping burden. Whether this is specific to our data set or holds true for other study populations needs to be evaluated further. It should be noted that all further analyses are based on the 13-SNPs-GRS.

**Table 3:** Model fit and discriminative accuracy of parsimonious models.

Model^1^	Variants^2^	R^2^	AUC
13-SNP model	*1,2,3,4,5,6,7,8,9,10,11,12,13*	0.2475	0.820
- TIMP3	*1,2,3,4,5,6,7,8,9,10,11,12*	0.2475	0.820
- PLA2G12A	*1,2,3,4,5,6,7,8,9,11,12*	0.2454	0.819
- APOE	*1,2,3,4,5,6,7,10,11,12*	0.2411	0.816
- LIPC^3^	*1,2,3,4,5,6,7,8,9,10*	0.2457	0.820

1 SNPs from one additional locus at a time were omitted from the 13-SNP model by starting with the locus with the smallest risk.

2 Numbering corresponds to IDs in Table 2.

3 This model contained the least number of SNPs without compromising R^2^ or AUC values.

### Distribution of the genetic risk score

**Figure 3:**
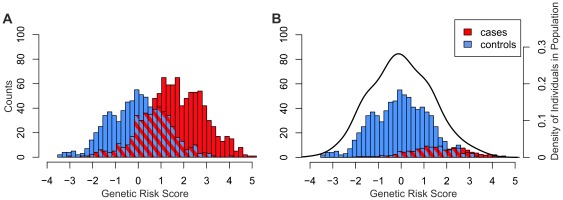
Genetic risk score distribution in the study population and in a modeled population. AMD cases are shown in red, controls in blue, while overlapping bars are shaded blue/red. (**A**) Genetic risk score distribution for cases (N = 986) and controls (N = 796) in the present study. (**B**) Counts of cases in (A) were scaled to represent 15% of the total population (assumed as AMD prevalence of the 85–90 year old general population). The density curve represents the risk score distribution in 381 European ancestry samples available through the 1000 Genomes Project (Release 20110521).

The distribution of GRS for cases and controls as observed in our study is given in **Figure 3A**. To provide a more realistic view of the GRS distribution, the proportion of cases were weighted to reflect a general distribution. For this modeling, an AMD prevalence of 15% was assumed as reported for the general population aged >85 years [30]–[32] (**Figure 3B**). The derived GRS is comparable to the distribution estimated from individual HapMap data (**Figure 3B**).

### Genetic risk score by age groups, gender and AMD subtype

We further investigated differences of the GRS between age-groups (below or older than 75 years), men and women, or types of AMD (GA, NV, or mixed GA+NV) using a significance level of 0.05/3 to account for the three subgroup tests performed.

Significant differences in mean GRS were found between younger (1.87, 95% CI: 1.69–2.05) and older (1.45, 95% CI: 1.36–1.54) AMD cases (p = 8.7×10^−5^), but there was no difference between the age-groups among controls (p = 0.18). The OR per GRS unit was 3.06 (95%CI: 2.64–3.59) for younger and 2.71 (95% CI: 2.44–3.05) for older individuals. We also found that the AUC restricted to the younger subjects (cases and controls) was higher (0.852) than when only older subjects (cases and controls) were included in the calculations (0.809).

Cases with mixed GA+NV had a significantly higher mean GRS (1.87, 95% CI: 1.69–2.04) compared to NV cases (1.44, 95% CI: 1.34–1.55, p = 6.6×10^−5^). It was also higher when compared to GA cases (1.65, 95% CI: 1.48–1.83, p = 0.03), although the latter was statistically not significant when applying a conservative Bonferroni-adjusted significance level of 0.05/3. The OR per GRS unit was also higher for mixed GA+NV cases (OR = 3.79, 95% CI: 3.13–4.67) than for NV cases (OR = 3.79, 95% CI: 3.13–4.67) or for GA cases (OR = 2.84, 95% CI: 2.44–3.33). This effect appeared to be independent of age, since mean age in GA (78.8 years, 95% CI: 77.9–79.6), NV (78.5 years, 95% CI: 77.9–79.0) and mixed GA+NV (79.4 years, 95% CI: 78.4–80.3) was similar. There was no significant difference in the GRS means between men and women neither among cases nor among controls.

These subgroup analyses demonstrate a higher genetic risk of the younger AMD patients compared to the older patients as well as a higher genetic risk for those with mixed late-stage manifestations (GA+NV) when compared to NV or GA alone.

### Genetic risk groups and relative risk estimates

To establish a classification scheme, we formed five equally sized intervals for the GRS spectrum (≤−1.79, (−1.79)–(−0.05), (−0.05)–1.70, 1.60–3.44, and >3.44; **Table 4**). The highest GRS category (no. 5) contained 7.92% of AMD cases, but only 0.13% of controls. In contrast, the two lowest GRS categories (nos. 1+2) jointly contained only 9.8% of cases, but 48.7% of controls. According to the HapMap data reflecting a general population, the proportion of subjects in the two lowest risk groups combined was 48.9% and 0.5% in the highest risk group. This is consistent with the general population being a mixture of mostly controls and only few cases.

**Table 4:** Five genetic risk groups and relative risk of AMD (ORs and 95% confidence intervals).

	Genetic risk groups				
GRS category	1	2	3	4	5
Sample size	N = 63	N = 417	N = 761	N = 450	N = 79
GRS interval	≤−1.79	]−1.79,−0.05]	]−0.05,1.70]	]1.70,3.44]	>3.44
Cases [%]	0.81	9.00	42.5	39.7	7.92
Cases <75 years [%]	1.70	6.20	34.0	42.3	15.8
Cases >75 years [%]	0.54	9.96	45.2	38.9	5.38
Controls [%]	6.99	41.7	43.6	7.50	0.13
Frequency in HapMap^1^	8.92	40.2	41.2	9.18	0.53
OR (95% CI)	0.12 (0.05–0.24)	0.22 (0.17–0.29)	reference	5.44 (4.02–7.46)	64.00 (14.11–1131.96)

GRS categories	low (1+2)	3	high (4+5)
Sample size	N = 480	N = 761	N = 529
GRS interval	≤−0.05	]−0.05,1.70]	>1.70
OR (95% CI)	0.21 (0.16–0.27)	reference	6.41 (4.76–8.76)
<75 years: OR (95% CI)	0.19 (0.10–0.33)	reference	12.66 (6.76–25.65)
> = 75 years: OR (95% CI)	0.22 (0.16–0.29)	reference	5.18 (3.70–7.38)

1 Fraction of individuals in 1000 Genome Project European Ancestry Samples residing in risk groups.

The relative risks were approximated as ORs for each GRS category using the middle category (no. 3) as reference (**Table 4**). It can be seen that the OR is dramatically increased for category four (OR = 5.44, 95% CI = 4.03–7.46) and even more for category five (OR = 64.00, 95% CI = 14.11–1132.96). The odds ratios are substantially decreased for categories two and one (OR = 0.22, CI = 0.17–0.29 and OR = 0.12, 95% CI = 0.05–0.24) compared to the reference. Thus, these GRS categories can effectively describe genetic risk groups for AMD.

Due to the substantial differences found in mean GRS for younger compared to older cases (see above), we derived these ORs also separately by age-group. To avoid scarce data, risk group one and two as well as four and five were combined to a low and a high risk group, respectively (**Table 4**). This highlights the higher genetic relative risk for AMD when restricting the analysis to the younger (OR = 12.66, 95% CI: 6.76–25.65) compared to the older (OR = 5.18, 95% CI: 3.70–7.38) subjects. Although the 95% confidence intervals overlap slightly, we observed a significant difference (p = 0.0194).

### Modeled absolute risk for late-stage AMD

To reflect the anticipated situation in the general population and to compute the absolute risk of AMD per GRS group, we computed the fraction of late-stage AMD cases per GRS category by (i) utilizing the fraction of cases and controls as observed in each GRS category (**Table 4**) and (ii) weighting the fraction of cases assuming various AMD prevalences (1%–15%). The fraction of cases and the fraction of subjects of the modeled general population (also for comparison in the HapMap sample) by GRS category are shown in **Table 5**. The fraction of late-stage AMD in the highest GRS group (absolute AMD risk) ranged from 38.6–91.7% depending on the assumed AMD prevalence which were chosen to correspond to the various age-groups as reported [30]–[32]. For example, in a general population with an AMD prevalence of 10% approximately 90% of the persons in the highest GRS group are expected to be affected by late-stage AMD. Consequently, the genetic relative risk for subjects in the highest GRS group (compared to the middle GRS group) is higher for younger compared to the older AMD cases. However, the absolute risk of AMD among subjects in the highest GRS group is higher for the older population due to the higher AMD prevalence among the older persons.

**Table 5:** Absolute risks for AMD by modeling a general population for various prevalences of AMD (reflecting various age-groups).

	Modeled prevalence (age-group [yrs])^1^	Absolute risk of AMD by genetic risk group [%]				
		1 (low)	2	3	4	5 (high)
GRS interval		≤−1.79	]−1.79,−0.05]	]−0.05,1.70]	]1.70,3.44]	>3.44
% cases, modeled general population						
	1% (65–69)	0.12	0.22	0.97	5.08	38.6
	2.5% (70–74)	0.30	0.55	2.44	12.0	61.5
	5% (75–79)	0.61	1.13	4.87	21.8	76.6
	10% (80–84)	1.30	2.40	9.80	37.0	87.4
	15% (>85)	2.00	3.70	14.7	48.3	91.7
% subjects, modeled general population						
	1%	6.84	40.9	43.6	8.31	0.32
	2.5%	6.68	40.1	43.8	8.50	0.34
	5%	6.69	40.1	43.6	9.12	0.52
	10%	6.38	38.5	43.5	10.7	0.91
	15%	6.10	36.8	43.5	12.3	1.30
% subjects, HapMap population^2^		8.92	40.2	41.2	9.18	0.53

1 Approximate age-groups corresponding to the modeled prevalences for 65 and 79 years [30], [31] and for those above 80 years [32].

2 see Table 4.

We again adopted the same cross-validation approach to compute absolute risks since the effect sizes of the variants in the GRS model, on which the absolute risk estimates are based, were estimated from our study data. This approach yielded overall similar estimates (**Table S2**).

## Discussion

Based on a genetic risk score including 13 reported SNPs from eight established AMD gene loci, we propose a five-category classification system that effectively differentiates subjects with high or low genetic risk. With this, we extend on earlier efforts to predict the genetic risk for late-stage AMD [26], [33]–[35]. Seddon et al. described a risk score model for six genetic variants in four loci also including environmental factors like BMI, smoking, age and diet (sample size was 1.446 individuals of which 279 progressed to AMD) [26]. Similarly, a study from Gibson et al. included 470 cases and 470 controls and reported an AUC of 0.83 (95% CI 0.81 to 0.86) using six SNPs in four loci and two environmental factors [33]. A study by Spencer et al. investigated one variant in each of four loci as well as age and smoking as environmental factors and found an AUC of 0.84 (95% CI: 0.81–0.88) [35]. Jakobsdottir et al. reported an AUC of 0.79 based on one SNP in each of three loci [34]. This study consisted of around 1.000 family-based cases and 429 controls as well as a case-control study with 187 cases and 168 controls. We evaluated 13 SNPs from 8 AMD loci in a well characterized and well powered case-control study and observed an AUC of 0.820, which is sufficient to classify AMD patients and controls into high risk and low risk groups [24]. Our study has not contributed to the identification of any of the 13 SNPs as AMD risk-increasing variants and would thus not be subject to winner's curse regarding the effect size. To our knowledge, this is a first study to include most of the currently known genetic loci for their value to predict late-stage AMD risk in a study that is independent of the identification of any of these loci.

Interestingly, we find a higher relative risk of the CFB SNP rs4151667 compared to CFH and ARMS2/HTRA1 risk-increasing SNPs particularly in the multivariable logistic regression model. This can also be seen in a previously published study (Seddon et al., Table 4) [36], although it needs to be noted that the models used in our and the published study differ in the sense that ours considers exclusively genetic factors while the other work largely focused on non-genetic factors. The smaller allele frequency of the CFB SNP (1.8% in our cases, 6.7% in the European ancestry 1000G individuals) compared to SNP frequencies in CFH and ARMS2/HTRA1 results in a reduced power to detect association and may explain why CFB SNP rs4151667 was not among those detected first by AMD GWAS.

As expected, the mean GRS was significantly higher in cases when compared to controls. Importantly, patients with late-stage AMD diagnosed at an earlier age had a significantly higher mean GRS than individuals that developed AMD later in life. This strongly suggests that genetic predisposition influences disease onset, which is also reflected in the higher relative AMD risk for younger subjects with an OR of 12.66 (95% CI: 6.76–25.65) when compared to older individuals with an OR of 5.18 (95% CI: 3.70–7.38). The mean genetic risk score in our control group was slightly lower but similar to the mean score in the HapMap sample (including a total of 381 European subjects from CEU, GBR, IBS, TSI and FIN, 1000 Genomes Project (Release 20110521, http://www.1000genomes.org, accessed 2 May 2012).). The slight discrepancy would be in-line with the fact that our controls were specifically selected to reveal no signs of early or late-stage AMD.

Limitations of our study for risk prediction should be acknowledged. First, the analysis was based on a case-control study, which has no element of a prospective study or a nested case-control study. The controls were often spouses of AMD patients and thus non-genetic risk factors could not be studied due to the known similarities among spouses regarding life style factors. However, our AMD patients were virtually incident AMD cases and thus the age at study entry is likely the age-at-diagnosis and the best possible proxy for age-of-onset (allowing for a delay of about 1–2 years between onset and diagnosis). In a case-control setting, absolute risk or positive/negative predictive values cannot be derived without making assumptions on the overall AMD prevalence, which a prospective cohort study could estimate directly. Thus, the predictive ability of the risk score groups greatly depends on those assumptions. Second, it might be considered a limitation but also a strength that our study included exclusively late-stage AMD with NV or GA in one or both eyes as well as highly-matched controls with no signs of early or late-stage AMD in any eye. A strength as our data might exhibit less disease misclassification than other studies, but a limitation as the genetic relative risk could be overestimated if the genetic risk is larger for subjects with both eyes affected than for those with only one affected eye. Third, we had no independent and equally well characterized data set available to separate model building from testing although this is also the case for all other studies published on AMD risk score model building [26], [33], [34]. Only one study [35] reported a small replication study. We avoided selecting SNPs for our model based on association signals in our own data but rather selected SNPs from the literature. However, the SNP-specific effect sizes utilized as weights in the genetic risk score computation were still estimated in our data set. Thus, estimations of AUC or absolute risk in the same data could lead to a slight over-estimation of risk. We therefore adopted a cross-validation approach as sensitivity analysis, which did not provide evidence of remarkable over-estimation.

The highest genetic risk group of our proposed five-category classification scheme can effectively identify subjects at high risk for AMD. The specificity in this risk group was 99.9% (95% CI: 99.3%–100%). For example, our data and model suggest that 87.4% of subjects testing positive at some time in life for a high genetic risk are likely to develop AMD in their mid-eighties (positive predictive value). Thus, this group of individuals could greatly profit from a sight-saving prevention or early intervention program while only 13% of (false-positive) subjects would be alarmed and treated unnecessarily. However, still a large number of cases would be missed if this was established as a screening method (sensitivity 8.0% (95% CI: 6.5%–9.9%), i.e. 92% of all AMD cases would not be found in the highest risk group). Also individuals in the second highest risk group could possibly profit from early intervention, which would increase sensitivity to 47.6% and decrease specificity to 91.2%. However, this would only be acceptable, if the prevention/intervention is not harmful to the 59.9% of subjects treated and alarmed unnecessarily (40.1% positive predictive value). These numbers are well in the range of established screening tests, e.g. for prostate cancer by prostate specific antigen (PSA) (positive predictive value = 25.1%, sensitivity = 72.1%, specificity = 93.2%, [37]), albeit with a higher predictive value at the cost of reduced sensitivity. Abnormal levels of PSA are detected in about 10% of the male population, which is comparable to the coverage of high risk group four and five [37]. Offering an effective prevention program to individuals in the highest AMD risk group (approximately 400,000 individuals in Germany alone), almost 10% of incident late-stage AMD could be avoided. If individuals in risk groups four and five are included (about 10% of the general population), up to 50% of future AMD patients could be addressed.

So far, only the progression of the neovascular complications in AMD can be slowed by treatment [38]. If disease progression to an advanced neovascular form is detected early in high risk patients, immediate intervention might prove essential to sustain full vision for a more extended time. Accordingly, high risk individuals could be advised to seek clinical follow-ups more frequently and could also benefit from dietary recommendations, including the intake of antioxidants [39] or omega-3 fatty acids [40], [41]. Identification of individuals at high risk for developing AMD may also help to include defined candidates in clinical AMD trials and thus may allow a better assessment of therapeutic effects.

In conclusion, our study provides a genetic risk score for late-stage AMD from a well characterized case-control study emphasizing the large proportion of disease explained by genetic markers particularly for younger subjects. We propose a classification scheme to identify subjects at high or low genetic risk that might be suitable for risk stratification in therapy studies or genetic screening once preventive treatment is available.

## Methods

### Ethics statement

This study followed the tenets of the declaration of Helsinki and was approved by the Ethics Review Board at the University of Würzburg, Germany. Informed written consent was obtained from each patient after explanation of the nature and possible consequences of the study.

### The study subjects

The case-control sample includes 986 AMD patients and 796 controls recruited from the Lower Frankonian area at the University Eye Clinic of Würzburg, Germany [14]. Controls were often unaffected spouses or nonrelated acquaintances of cases of similar age as the patient. All patients and controls were examined by a trained ophthalmologist (CNK). Stereo fundus photographs were graded according to standardized classification systems as described previously [9], [42], [43]. Only patients with severe forms of AMD (GA or NV) in at least one eye and signs of early AMD (e.g. large soft drusen) in the other eye were included. The patients were divided into three subgroups according to their type of late-stage AMD: patients with GA in the severe eye, patients with NV in the severe eye and patients that had either GA in one eye and NV in the other eye or that showed both late-stage forms in the same eye (mixed GA+NV). Mean age in cases was 78.7 (±6.5) years and 78.3 (±5.1) in controls. A total of 34.1% of cases and 39.1% of controls were male. Study characteristics are summarized in **Table 1**.

### Genotyping

Genomic DNA was extracted from peripheral blood leukocytes according to established protocols. Genotyping of SNPs was achieved by direct sequencing, restriction enzyme digestion of PCR products, TaqMan SNP Genotyping (Applied Biosystems, Foster City, USA) or primer extension of multiplex PCR products with detection of the allele-specific extension products by the matrix-assisted laser desorption/ionization time of flight (MALDI-TOF) mass spectrometry method (Sequenom, San Diego, USA) (**Table S3**). Direct sequencing was performed with the Big Dye Terminator Cycle Sequencing Kit Version 1.1 (Applied Biosystems, Foster City, USA) according to the manufacturer's instructions. Reactions were analyzed with an ABI Prism Model 3130xl Sequencer (Applied Biosystems). TaqMan Pre-Designed SNP Genotyping Assays (Applied Biosystems) were performed according to the manufacturer's instructions. Additionally, some variants were genotyped by PCR followed by restriction enzyme digestion (New England Biolabs, Ipswich, USA) and subsequent restriction fragment length analysis. The c.del443ins54 variant in the 3′-region of the *ARMS2* locus was genotyped by a single PCR with oligonucleotide primers 5′-ACTCATCACGTCATCACCAAT-3′ and 5′-CTCTCTGCAGCCCTCATTTG-3′ resulting in distinct fragment sizes due to the presence or absence of the deletion/insertion polymorphism.

### Estimating genetic risk and model fit

Genotypes were coded as the number of AMD risk increasing alleles (0, 1, and 2). Logistic regression analyses were carried out using the R software [44]. Odds ratios (OR) per risk allele and 95% confidence intervals (95% CI) were calculated from the estimated beta-coefficients to derive an approximate relative risk. The goodness-of-fit of each model was assessed by calculating McFaddens pseudo R^2^ [45], which however, does not reflect the variance explained by the model [46].

### Computing the genetic risk score

Based on the intercept “a” and the single-SNP beta-coefficients estimated using the logistic regression model including all SNPs at once, the genetic risk score (GRS) was calculated as
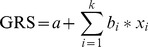
(1)with k being the number of SNPs in the model and x_i_ the genotype of the ith SNP. Here, “a” denotes a constant that centers the risk score distribution around zero and b_i_ relates to the ith variant. The odds ratio of the effect of the ith variant is thus given by exp(b_i_) [19], [23], [26]. The mean GRS by age-group, sex, or AMD subtype were compared based on the independent samples t test using the R software [44] and differences were considered as significant, if P<0.05/3 accounting for the three comparisons performed.

### Assessing the discriminative ability

To estimate the ability of a potential genetic screening test to discriminate between AMD cases and healthy subjects, we computed the receiver-operating-characteristic (ROC) curve. This involves ranking all subjects according to their GRS starting with the smallest, computing sensitivity and specificity at each possible GRS cut-off, and plotting sensitivity versus 1-specificity. The area-under-the-curve (AUC) is a measure of how well the GRS cut-offs can separate AMD cases from controls. We used the package EPICALC [47] for AUC computations and forest plots were generated with RMETA [48].

### Internal validation by cross-validation

Although we have not selected the SNPs into the model based on their association in our data set but rather with information from the literature, there is a potential overestimation of the AUC due to the fact that we used the SNP effect sizes to weigh the risk alleles when computing the GRS. Thus, we conducted a sensitivity analysis using a cross-validation approach to derive AUC estimates that are not subject to this bias to compare with the original data AUC. We randomly assigned 2/3^rd^ of the data to the model building (to compute the effect sizes and thus establish the GRS model) and 1/3^rd^ of the data to testing (to compute the AUC and positive predictive values) ([49], [50]). We repeated this 2000 times and computed the average AUC as an unbiased estimate.

### Modeling of the absolute risk by GRS group

In order to derive the fraction of cases in the five GRS categories as expected in the general population (corresponding to the absolute AMD risk) from the number of cases (N_cases = 986) and controls (N_controls = 796) in our case-control study, we weighted the number of AMD cases in our study by
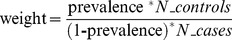
(2)where prevalence denotes the fraction of AMD cases in the general population, that we chose to reflect previously reported prevalences of AMD in the various age groups (65–69 years: 1%, 70–74 years: 2.5%, 75–79 years: 5%, 80–84 years: 10% and >85 years: 15%) [30]–[32]. These were also used to compute positive and negative predictive value for the highest GRS category as a screening test for AMD. The cross-validation approach described above was also adopted for a sensitivity analysis to compute unbiased absolute risk.

## Supporting Information

Figure S1 **Risk estimates for 16 AMD associated variants by disease subtypes.** Logistic regression models were fitted with all patients (N = 986), GA cases only (N = 229), NV cases only (N = 581) or mixed GA+NV cases (N = 176) versus controls (N = 796). Odds ratio estimates (OR) are given per risk allele; horizontal bars indicate 95% confidence intervals and the arrow indicates that the boundary extends below 1 or above 6.
(TIF)

Table S1 **Published genetic variations associated with AMD.**
(DOC)

Table S2 **Cross validated absolute risks for late stage AMD in different risk groups in the modeled population.**
(DOC)

Table S3 **Primers and methods used for genotyping.**
(DOC)
