# Supplementary figures and images for: Modelling the Genetic Risk in Age-Related Macular Degeneration
Source: PLoS One. 2012 May 30;7(5):e37979. doi: 10.1371/journal.pone.0037979 (PMC3364197; doi:10.1371/journal.pone.0037979)

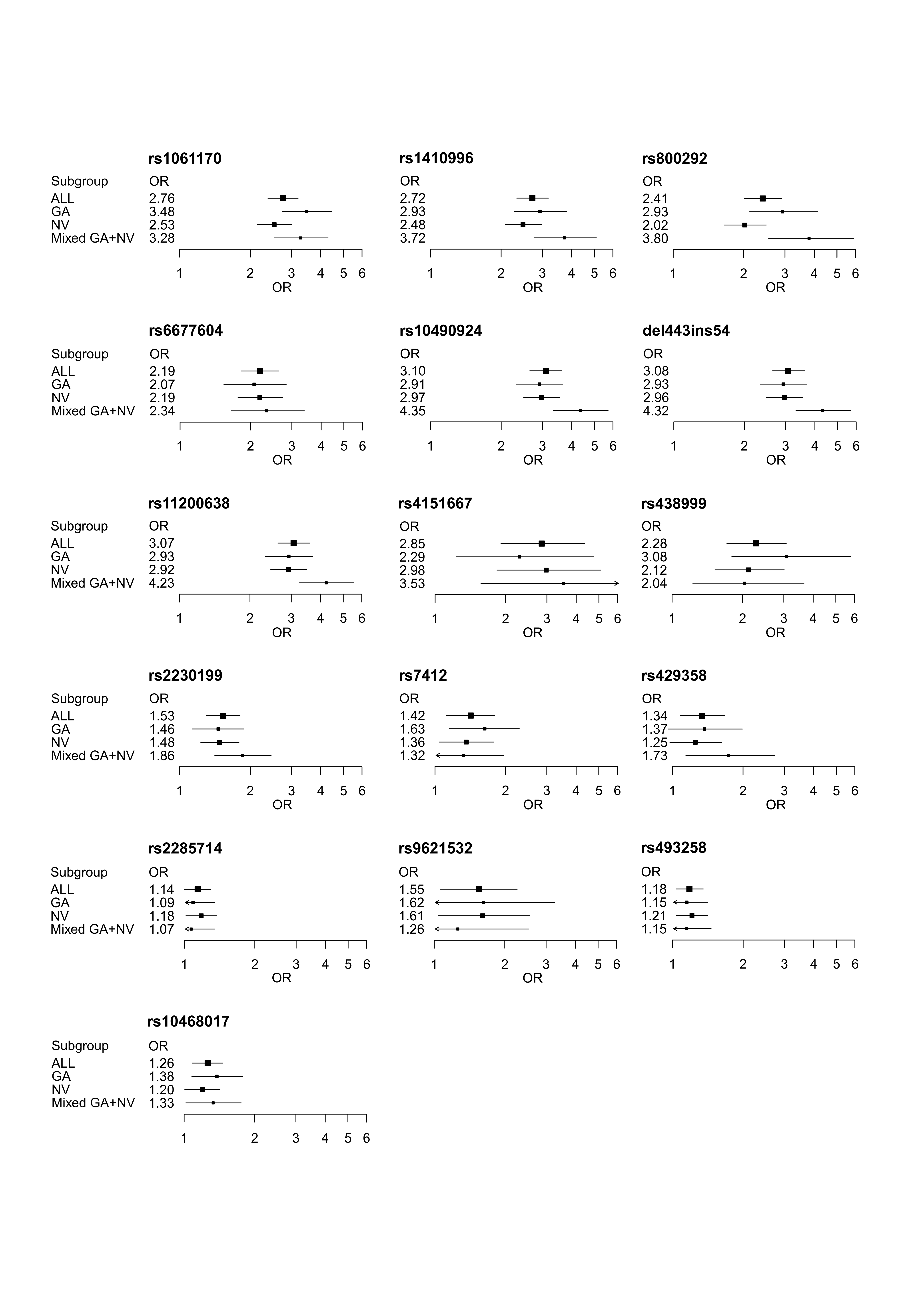

Supplement: Figure S1 — Risk estimates for 16 AMD associated variants by disease subtypes. Logistic regression models were fitted with all patients (N = 986), GA cases only (N = 229), NV cases only (N = 581) or mixed GA+NV cases (N = 176) versus controls (N = 796). Odds ratio estimates (OR) are given per risk allele; horizontal bars indicate 95% confidence intervals and the arrow indicates that the boundary extends below 1 or above 6. (TIF) [file pone.0037979.s001.tif]
