# Supplementary material for: Modelling the Genetic Risk in Age-Related Macular Degeneration
Source: PLoS One. 2012 May 30;7(5):e37979. doi: 10.1371/journal.pone.0037979 (PMC3364197; doi:10.1371/journal.pone.0037979)
Supplement: Table S1 — Published genetic variations associated with AMD. (DOC) [file pone.0037979.s002.doc]

**Supporting Table 1: Published genetic variations associated with AMD**

| **Variant** | **Gene** | **Year** | **Ref.** |
| --- | --- | --- | --- |
| rs1061170 | *CFH* | 2005 | [8] |
| rs2274700 (proxy: rs1410996) | *CFH* | 2006 | [10,13] |
| rs800292 | *CFH* | 2005 | [9] |
| ∆CFHR3/CFHR1 (proxy: rs6677604) | *CFH/CFHR* | 2007 | [11–13] |
| rs10490924 | *ARMS2* | 2005 | [7] |
| c.del443ins54 | *ARMS2* | 2008 | [48] |
| rs11200638 | *HTRA1* | 2006 | [49] |
| rs4151667 | *CFB* | 2006 | [15] |
| rs547154 (proxy: rs438999) | *CFB* | 2006 | [15] |
| rs2230199 | *C3* | 2008 | [50] |
| rs7412 | *APOE* | 1998 | [21,22] |
| rs429358 | *APOE* | 1998 | [21,22] |
| rs2285714 | *CFI/PLA2G12A* | 2009 | [18] |
| rs493258 | *LIPC* | 2010 | [19] |
| rs10468017 | *LIPC* | 2010 | [20] |
| rs9621532 | *SYN3/TIMP3* | 2010 | [19] |
