# Supplementary material for: Modelling the Genetic Risk in Age-Related Macular Degeneration
Source: PLoS One. 2012 May 30;7(5):e37979. doi: 10.1371/journal.pone.0037979 (PMC3364197; doi:10.1371/journal.pone.0037979)
Supplement: Table S2 — Cross validated absolute risks for late stage AMD in different risk groups in the modeled population. (DOC) [file pone.0037979.s003.doc]

**Supporting Table 2. Cross validated absolute risks for late stage AMD in different risk groups in the modeled population**

|  | Modeled prevalence (age group in yrs)^1^ | **Positive predictive value in risk group [%]** | | | | |
| --- | --- | --- | --- | --- | --- | --- |
|  |  | **1 (low)** | **2** | **3** | **4** | **5 (high)** |
| **Fraction of cases in the modeled general population (absolute risk) [%]** |  |  |  |  |  |  |
|  | 1% (65-69) | 0.13 | 0.22 | 0.91 | 4.22 | 46.8 |
|  | 2.5% (70-74) | 0.35 | 0.56 | 2.26 | 10.0 | 58.1 |
|  | 5% (75-79) | 0.70 | 1.13 | 4.54 | 18.5 | 68.4 |
|  | 10% (80-84) | 1.46 | 2.39 | 9.13 | 32.4 | 80.0 |
|  | 15% (>85) | 2.20 | 3.72 | 13.7 | 42.7 | 85.6 |
| **Fraction of cases and controls in our study^2^** |  |  |  |  |  |  |
| Cases [%] |  | 1.00 | 8.50 | 38.7 | 40.8 | 11.0 |
| Controls [%] |  | 7.90 | 38.2 | 43.5 | 10.2 | 0.40 |

^1^ Approximate age groups corresponding to prevalences according to [31,32] for prevalences between 65 and 79 years and [33] for prevalences above 80 years

^2^ Averaged fractions of controls and cases observed in 2,000 test sets in each risk group
