# Supplementary material for: Modelling the Genetic Risk in Age-Related Macular Degeneration
Source: PLoS One. 2012 May 30;7(5):e37979. doi: 10.1371/journal.pone.0037979 (PMC3364197; doi:10.1371/journal.pone.0037979)
Supplement: Table S3 — Primers and methods used for genotyping. (DOC) [file pone.0037979.s004.doc]

**Supporting Table 3**: Primers and methods used for genotyping

| **Nearby gene(s)** | **Marker** | **Method** | **Forward primer (5‘-3‘)** | **Rreverse primer (5‘-3‘)** | **Universal Extension primer (MALDI-TOF)** |
| --- | --- | --- | --- | --- | --- |
| *CFH* | rs1061170 | Sequencing | TTGCACACAAGATGGATGGT | GCATCTGGGAGTAGGAGACC |  |
|  |  | RFLP (Tsp509I)^1^ | CCTTTGTTAGTAACTTTAGTTCGTCTT | CCAAAAACTAAATAGGTCCATTGGT |  |
|  | rs1410996 | MALDI-TOF | ACGTTGGATGCCGTCAATGAGATTTACGTC | ACGTTGGATGCCTCTACATCAGTGGTATAG | ACGCAGTCCCTGACTACCTCATG |
|  | rs800292 | Taqman (C___2530382_10) |  |  |  |
|  |  | MALDI-TOF | ACGTTGGATGAAATGCCGCCCTGGATATAG | ACGTTGGATGTAAGAGCAACCCATTCTCCC | CCTGGATATAGATCTCTTGGAAAT |
|  | rs6677604 | Sequencing | TTGCTTGAGGAAAGTTGTGC | TTCCCATCTCCTGTCACAAA |  |
|  |  | MALDI-TOF | ACGTTGGATGCCACCAAAGCACAATACCTC | ACGTTGGATGCATGACTTACATAGTTGCCC | TAGCTGTGAGTCCTTTCC |
| *ARMS2/HTRA* | rs10490924 | Sequencing | GCCTGAGATGGCAAGTCTGT | TGTAGCAGGTGCATTGGAAG |  |
|  | del443ins54 | PCR² | ACTCATCACGTCATCACCAAT | CTCTCTGCAGCCCTCATTTG^3^ |  |
|  | rs11200638 | RFLP (EagI)^1^ | ATGCCACCCACAACAACTTT | GGTTCTCTCGCTGAGATTCG |  |
| *CFB* | rs4151667 | Sequencing | GGTCTGGAGTTTCAGCTTGG | TCTTGGAGAAGTCGGAAGGA |  |
|  |  | MALDI-TOF | ACGTTGGATGCTTCTCTCCTGCCTTCCAAC | ACGTTGGATGCAAGAGGCCCAAGATAAAGG | ATCTCAGCCCCCAAC |
|  | rs438999 | Sequencing | GAGGTCAGGGGTCATGAGAA | AGACAGGGATTCATGGGATG |  |
|  |  |  | ACGTTGGATGACAACCTCCTTGTCTCTTCG | ACGTTGGATGAAGAGTCACCTGGCCAGAAG | GGGGACTTATGGGGAAATCCAACTC |
| *C3* | rs2230199 | RFLP (HhaI)^1^ | GTGGTTGACGGTGAAGATCC | CAAGATCCGGAAGCTGGAC |  |
|  |  | MALDI-TOF | ACGTTGGATGCAACAGGGAGTTCAAGTCAG | ACGTTGGATGTCCACCACTTGGGTCCCGAA | AGTGAGTTCAAGTCAGAAAAGGGG |
| *APOE* | rs7412 | Sequencing | GATGGACGAGACCATGAAGG | CTCGAACCAGCTCTTGAGG |  |
|  | rs429358 | Sequencing | GATGGACGAGACCATGAAGG | CTCGAACCAGCTCTTGAGG |  |
| *PLA2G12A* | rs2285714 | Sequencing | CAAGCCACCAGATCATCCTT | CAAATGCCTTTTGCAGCTTA |  |
|  |  | MALDI-TOF | ACGTTGGATGCCTGACAAAGTGTTGCAACC | ACGTTGGATGCAGTCATTCTTGCTTTTGCC | GGGTGACACGACAGGTGCTATGA |
| *SYN3* | rs9621532 | RFLP (EcoO109I) | GGTTCTACTGGCTGGGTGAA | TACCCCCACTACCCCTAGTT |  |
|  |  | MALDI-TOF | ACGTTGGATGTGAAAGGGATTGAAAGCAGG | ACGTTGGATGTCTGGGCAGCCTGAAAACTC | GGGATTGAAAGCAGGTCATTA |
| *LIPC* | rs10468017 | RFLP (SspI) | TTTACGGTCTCCAAGACTGCT | CCAAGTTCATTCACAGGGACT |  |
|  | rs493258 | Taqman ( C___1929355_10) |  |  |  |
|  |  | Sequencing | AGACCAGCAGGCATCACC | CCAGAAACAAACAAGTGGAGTG |  |

^1^ Restriction enzyme used for RFLP assay

^2^ PCR with these primers yielded two distinct PCR products of different lengths corresponding to each allele.

^3^ SNPs were found in primer binding site
